# Efficacy of a Multicomponent Nutraceutical Formulation for the Prevention and Treatment of Urinary Tract Stones

**DOI:** 10.3390/ijms24098316

**Published:** 2023-05-05

**Authors:** Maria Maisto, Elisabetta Schiano, Gianni Luccheo, Luigi Luccheo, Ernesto Alfieri, Vincenzo Piccolo, Fortuna Iannuzzo, Ritamaria Di Lorenzo, Gian Carlo Tenore

**Affiliations:** 1 Department of Pharmacy, University of Naples Federico II, Via Domenico Montesano 59, 80131 Naples, Italy; maria.maisto@unina.it (M.M.); vincenzo.piccolo3@unina.it (V.P.); fortuna.iannuzzo@unina.it (F.I.); ritamaria.dilorenzo@unina.it (R.D.L.); giancarlo.tenore@unina.it (G.C.T.); 2 Nutriphyt Lab, Anvest Health s.r.l., Via Gabriele Camozzi 12, 20152 Milan, Italy; g.luccheo@anvesthealth.com (G.L.); l.luccheo@anvesthealth.com (L.L.); e.alfieri@anvesthealth.com (E.A.)

**Keywords:** nutraceutical formulation, myorelaxant effect, anti-edema activity, calcium-oxalate crystal, urinary tract stones, uric acid cellular transporters

## Abstract

Urolithiasis is a complex and multifactorial disease characterized by the formation of calculi at the urinary tract level. Conventional therapeutic prophylaxis relies on the use of Ca-blockers, alkalis, diuretics, and anti-edema agents, but their prolonged utilization is often limited by several side effects. In this scenario, the aim of the present work was the design of an innovative multi-component nutraceutical formulation (NF) for the management of urinary stones consisting of a synergistic combination of natural aqueous extracts of *Oreganum vulgare* L. (1% of saponin), *Urtica dioica* (0.8% of β-sitosterol), *Phyllanthus niruri* (15% of tannins *w*/*w*), and *Ceterach officinarum* in association with bromelain, K, and Mg citrate. To assess the potential of NF also in the treatment of uric acid (UA) stones, the effects on the expression of the cellular UA transporters OAT1 and URAT1 were investigated in a renal tubular cell line. In addition, the myorelaxant effect of NF was investigated in a human pulmonary artery smooth muscle cell (HPASMC) model resulting in a decreased muscle contractility of −49.4% (*p* < 0.01) compared to the control. The treatment with NF also showed a valuable inhibition of in vitro calcium-oxalate crystal formation, both in prevention (−52.3% vs. control, *p* < 0.01) and treatment (−70.8% vs. control, *p* < 0.01) experiments. Finally, an ischemic reperfusion rat model was used to evaluate the NF anti-edema effects, resulting in a reduction in the edema-related vascular permeability (Normalized Gray Levels, NGL = 0.40 ± 0.09, *p* < 0.01, −67.1% vs. untreated rats). In conclusion, the present NF has shown to be a promising natural alternative for managing urinary tract stones.

## 1. Introduction

Urolithiasis is one of the oldest known and widespread pathological condition of the urinary tract affecting 5% of the population worldwide every year [1]. Generally, the formation of urinary tract stones (UTS) is a complex and multifactorial process that can be described by three consecutive steps, including crystal aggregation, nucleation, and growth of insoluble particles [2]. The UTS could have different chemical compositions. Specifically, calcium-containing stones are the most diffuse kidney stones (75–90%), followed by magnesium ammonium phosphate (struvite) (10–15%), uric acid (3–10%), and cystine (0.5–1%) stones [3,4]. Clinically, the passage of kidney stones into the urinary tract can cause several symptoms, such as obstruction, infection, pain, and hemorrhage [3]. Over the last decades, the management of this condition has changed drastically. On one side, the laparoscopy surgery approach aims to completely remove kidney stones, although these procedures are complicated, relatively expensive, and do not prevent the recurrence of stones [5]. On the other side, conventional therapeutic treatment mainly aims at preventing and alleviating symptoms. Since stone formation is a multifactorial process, therapeutic prophylaxis could rely on several agents that can specifically act on the different steps of calculi formation. In particular, the stone expulsive approach, also known as medical expulsion therapy (MET), is based on treatment with calcium-channel blockers such as nifedipine, which are supposed to relax the urethral smooth muscles, thus favoring spontaneous stone excretion [6]. This type of treatment is often combined with an anti-edema agent to facilitate the passage of the ureteral stone [7]. Other therapeutic approaches conventionally used to prevent hypercalciuria and hyperoxaluria (major causes of calculi formation) include the use of diuretic agents, such as thiazides aimed to reduce urinary calcium concentration [8,9]. Additionally, alkali-citrate is widely employed in pharmaceutical practice for the prevention of urolithiasis, primarily due to its ability to sequester calcium in the urine by forming a Ca–citrate complex. Specifically, the citrate–calcium complex is more soluble than the oxalate-calcium complex and thus directly inhibits crystal agglomeration and subsequent precipitation [10]. The pharmacological approach to the treatment of urate-based stones has been somewhat different. Currently, several drugs are available to lower uric acid (UA) levels, such as allopurinol, but their efficacy has been severely limited by proven adverse effects, including headaches, allergies, skin rashes, increased aminotransferase, and nephritis [11].

Overall, all the above-mentioned pharmacological approaches are characterized by low effectiveness and tolerability, and their utilization is often limited by the various adverse effects described previously, such as hyperkalemia, acidosis, etc [12]. Therefore, due to the complexity and low compliance of surgical techniques and the low efficacy of conventional drug remedies, there is an urgent need to identify innovative therapeutic alternatives for managing urolithiasis. Traditionally, various plants have been widely utilized as therapeutic remedies for treating some urinary tract disorders, including urolithiasis [13]. Specifically, two peculiar plant species, namely *Ceterach officinarum* Willd. (CE) (belonging to the *Asplenium* family) and *Phillanto niuri* (PN) (belonging to the *Euphorbiaceae* family), are generally recognized as “stonebreaker“ plants, because of their strong activity on urinary stones [14]. From the molecular point of view, regarding *CE,* it was reported that its peculiar flavonoid composition was able to promote only the nucleation of small calcium oxalate crystals, without inducing their aggregation, leading to the formation of small crystals which are spontaneously eliminated with the urine avoiding their deposition at the urinary tract level [15]. While regarding PN, several pieces of evidence seem to indicate its relevant interference in the urinary calculi biomineralization process by promoting an interaction between the crystal and the macromolecules of PN organic components [16]. Nevertheless, the molecular mediators of such effects are not yet identified [16]. While regarding the PN modulatory activity on UA clearance, phyllanthin and hypophyllanthin [17], two representative PN lignans, have shown valuable uricosuric activity in hyperuricemic rats [18].

In this scenario, the main goal of the present work was to formulate an innovative nutraceutical formulation (NF) based on the synergistic combination of different components, some of which have a traditional and consolidated use in litholysis therapy, i.e., extracts of PN and CE, potassium (K), magnesium (Mg) citrate, which are commonly considered stone inhibitors (SI), in association with other natural agents with less documented effects in urolithiasis management, i.e., ortica (*Urtica dioica*) aqueous extract (OE), origanum (*Origanum vulgare*) aqueous extract (OrE), and bromelain. In order to evaluate the potential of NF in inhibiting the formation of UA stones formation, the modulatory activity on UA clearance was investigated in human renal tubular epithelial cell lines. Moreover, the inhibition of NF on Calcium oxalate (CaOx) stone formation and growth was evaluated in vitro. Finally, the muscle-relaxing effect of NF was evaluated in a smooth muscle cell line to assess its applicability in MET therapy, while the anti-edema activity of NF was investigated in an ischemic reperfusion rat model.

## 2. Results

### 2.1. Modulatory Activity on UA Clearance

**Figure 1:**
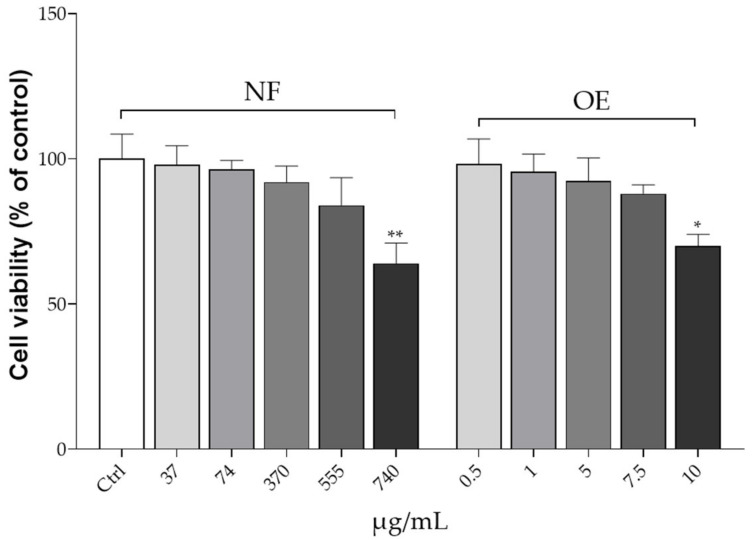
MTT analysis in HK2 cell line after 48 h incubation with different concentrations of the testing samples. Values are presented as means ± S.D. of four replicates. Data were analyzed with one-way ANOVA followed by Dunnett’s post hoc test; * *p* ≤ 0.05, ** *p* ≤ 0.01, significantly different than the Ctrl group. Abbreviations: Ctrl, control; NF, nutraceutical formulation; OE, Ortica extract.

To evaluate the effect of both NF and OE (as reference samples) on the expression of the transporters OAT1 and URAT1 in the HK2 cell line, RT-qPCR was performed. Notably, these cellular transporters are generally recognized as key potential therapeutic targets for hyperuricemia, as 70% of urate in humans is regulated by this renal urate transport system [19]. As the first aim, we investigated whether NF or OE could exert cytotoxic effects in the HK2 cell line. Cells were treated with increased concentrations of NF (37, 74, 370, 555, and 740 µg/mL) and OE (0.5, 1, 5, 7.5, and 10 µg/mL) for 48 h, and cell viability was assessed by MTT test. The results of cell viability after treatment with the test samples are shown in Figure 1.

**Figure 2:**
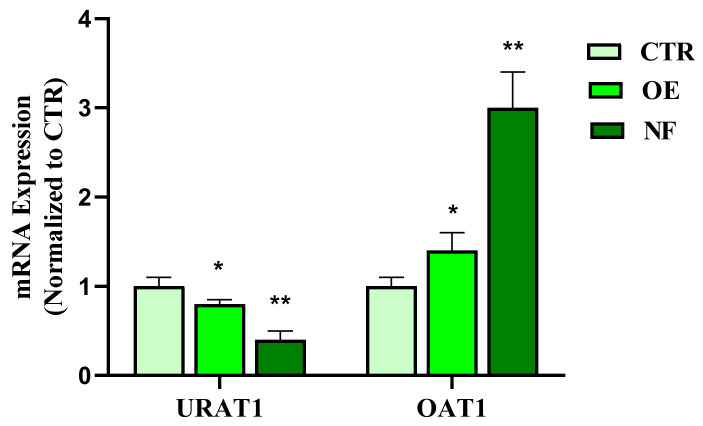
Effects of treatment with NF and OE on mRNA URAT1 and OAT1 expression in HK2 cells. Expression of URAT1 and OAT1 was assessed with qPCR in HK2 cells upon 6 h of treatment with OE (5 µg/mL) and NF (370 µg/mL). Data were shown as mean ± SD of at least three independent experiments (* *p* < 0.05 and ** *p* < 0.01 vs. CTR).

Evidently, cells exposed at a concentration of 740 µg/mL and 10 µg/mL showed a significant reduction in cell survival in the NF (*p* < 0.01 vs. Ctrl) or the OE sample (*p* < 0.05 vs. Ctrl), respectively, whereas concentrations below these values were found to be safe. Therefore, based on the outcomes of this experiment, intermediate concentrations of 370 µg/mL and 5 µg/mL for the NF and OE samples, respectively, were selected for further experiments involving the investigation of the effects on URAT1 and OAT1 mRNA expression. In this regard, our data indicate that OE causes a significant 20% reduction in URAT1 mRNA expression (*p* < 0.05 vs. Ctrl), while treatment with the complete NF results in the highest URAT1 expression reduction (−60% vs. Ctrl, *p* < 0.01). The results are completely different when considering the effects of the same treatments on OAT1 expression. Incubation with OE alone led to a 40% increase in mRNA OAT1 expression (*p* < 0.01 vs. Ctrl), whereas treatment with NF resulted in a 200% increase in mRNA OAT1 expression (*p* < 0.01 vs. Ctrl). As expected, treatment with Ctrl did not produce any change in terms of URAT1 and OAT1 mRNA expression (Figure 2).

### 2.2. Muscle Relaxing Effects

**Figure 3:**
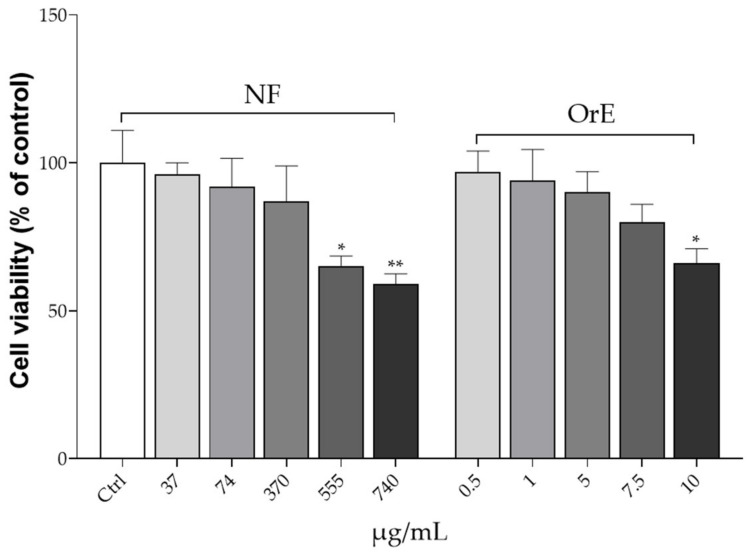
MTT analysis in HPASMC after 48 h of incubation with different concentrations of the testing samples. Values are presented as means ± S.D. of four replicates. Data were analyzed with one-way ANOVA followed by Dunnett’s post hoc test; * *p* ≤ 0.05, ** *p* ≤ 0.01, significantly different compared with the Ctrl group. Abbreviations: Ctrl, control; NF, nutraceutical formulation; OrE, Origanum extract.

As a preliminary step toward investigating the potential of NF as a muscle relaxant, the cytotoxic assessment was performed in the HPASMC by MTT test. Figure 3 shows results after 48 h treatment of HPASMC with different concentrations of NF (37, 74, 370, 555, and 740 µg/mL) and its reference sample OrE (0.5, 1, 5, 7.5, and 10 µg/mL). Similar to what was previously observed, incubation with the highest concentrations of the test samples resulted to be associated with a reduction in cell viability (*p* < 0.01 for 740 µg/mL NF, *p* < 0.05 for 555 µg/mL NF, *p* < 0.05 for 10 µg/mL OE sample, compared to Ctrl). Therefore, we decided to operate at concentrations below these values.

**Figure 4:**
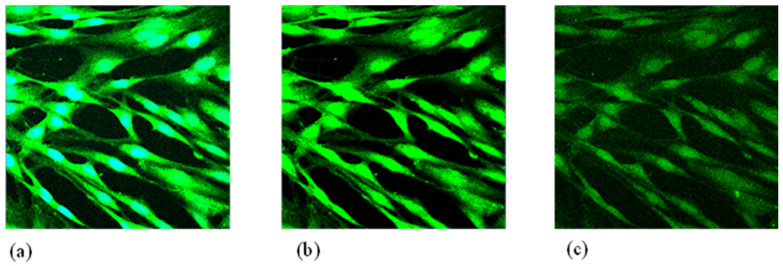
Intracellular Ca^2+^ fluorescence ([Ca^2+^]_i_) in cultured human pulmonary artery smooth muscle cells (HPASMC). Cells were cultured in confocal dishes and incubated with Fluo-3/AM (5 μM) at room temperature. (**a**) Control; (**b**) OrE; (**c**) NF. Abbreviations: Ctrl, control; NF, nutraceutical formulation; OrE, Origanum extract.

To test the potential of the tested NF to counteract muscle contraction caused by the occurrence of calculi in the distal ureter, and in particular to facilitate stone expulsion, the NF inhibitory activity on smooth muscle contractility in HPASMC was evaluated. In our model, muscle cell contractility was directly correlated with intracellular calcium concentration [Ca^2+^]_i_, which was monitored by the fluorescence method using fluo-3/AM (5 μM) as a fluorometric marker. Figure 4 shows the results obtained. In particular, Figure 4a shows how stimulation of these cells with ACh causes calcium to enter the cell, bringing the [Ca^2+^]_i_ to the concentration of 51.5 ± 8.9 nM (Ctrl sample). Figure 4b shows the same experimental system when cells were incubated with OrE, which resulted in a significant decrease in terms of fluorescence, corresponding to a decrease in [Ca^2+^]_i_ to a value of 43.8 ± 7.2 nM (−15.0%, *p* < 0.05 vs. Ctrl). Finally, incubation with the NF led to a greater decrease in fluorescence, corresponding to a reduction in [Ca^2+]^_i_ to a value of 25.7 ± 9.2 nM (−49.4%, *p* < 0.01 vs. Ctrl, Figure 4c).

### 2.3. In Vitro Inhibition of Calcium Oxalate (CaOx) Stone Formation

To determine the potential effects of the complete NF compared to the activity of some of its specific constituents, that are citrate K and Mg, PN, and CE, generally known as SI (stone inhibitors), an in vitro precipitation study of CaOx crystals was performed. Specifically, 3 mL of 0.1 M sodium oxalate per 50 mL of a synthetic urine solution was progressively added until the visible formation of CaOx crystals precipitates, which was achieved at 0.0402 g of added sodium oxalate in the control group, resulting in a pellet with the height of 141 mm. The same experimental protocol was repeated both with the addition of the complete NF and only with SI_._ Strikingly, our results show that the addition of SI agents alone inhibited the formation of CaOx crystals by −15.7% (*p* < 0.05 vs. Ctrl), while incubation with the NF resulted in a −52.3% decrease of CaOx crystals precipitation (*p* < 0.001 vs. Ctrl). These results suggest a potential preventive effect of NF on CaOx stone formation. Moreover, the same experiment was conducted to evaluate not only the preventive effect but also the potential therapeutic activity of the NF sample. Practically, the precipitation of CaOx crystals was first induced in the synthetic urine solution by the addition of sodium oxalate, resulting in a pellet formation of 141 cm in height. To this system, both the SI mix components and the NF were added separately and incubated for 30 min. After this incubation period, the pellet height decreased to 1.16 cm (−17.2%, *p* < 0.01 vs. Ctrl) after treatment with SI components alone, while pellet height decreased to 0.41 cm after incubation with NF (−70.8%, *p* < 0.01 vs. Ctrl). Overall, the results obtained indicate the higher ability of NF to inhibit crystal formation and dissolution compared to the SI mixture.

### 2.4. Evaluation of Anti-Edema Effects

**Figure 5:**
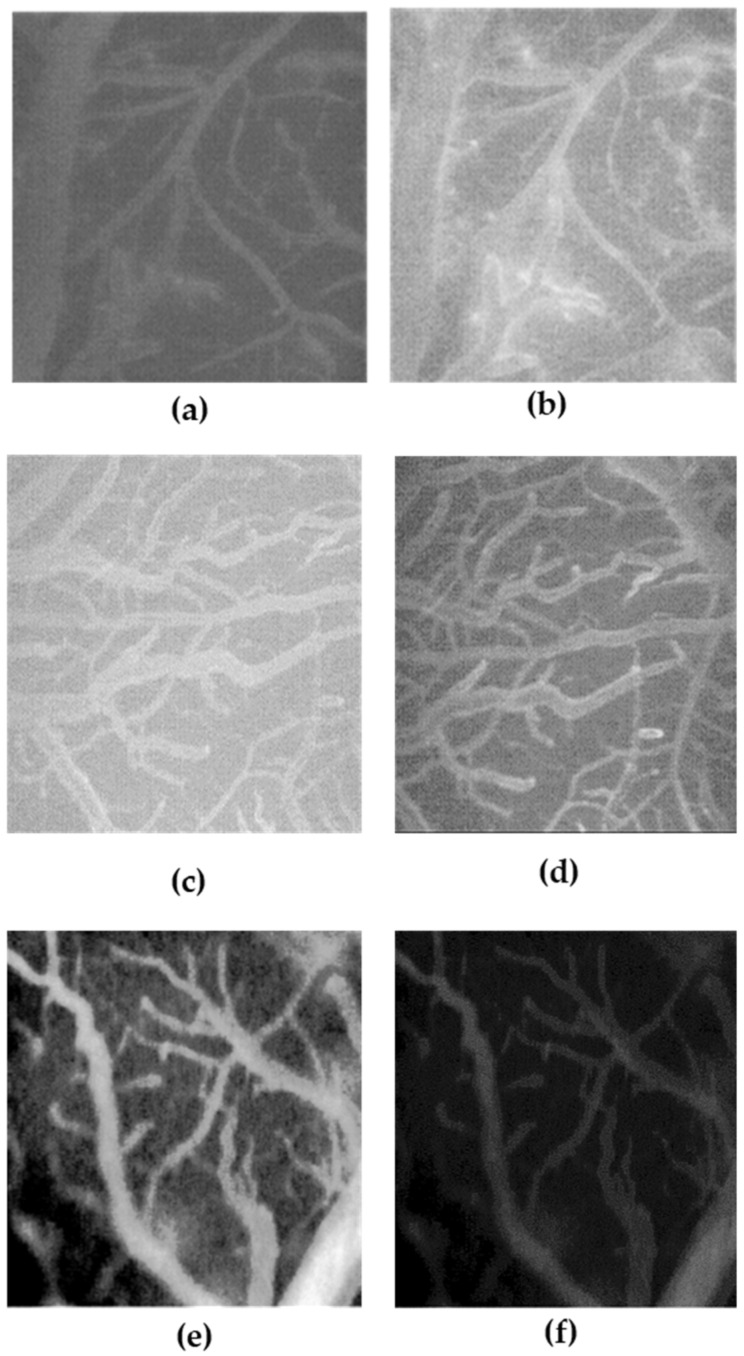
Arterial circulation and edematous effect before (**a**) and after (**b**) BCCAO in rats of the control group; Arterial circulation and edematous effect induced by BCCAO before (**c**) and after (**d**) intravenous administration of bromelain (1.8 mg/kg b.w.). Arterial circulation and edematous effect induced by BCCAO before (**e**) and after (**f**) intravenous administration of NF (33 mg/kg b.w.). Abbreviations: Ctrl, control; NF, nutraceutical formulation.

Urethral edema alleviation is a key factor in the treatment of urolithiasis. Edema plays a central role in arresting ureteral stone passage, so its reduction could facilitate stone expulsion [20]. For this reason, the anti-edema effects of bromelain alone (the only component contained in NF with described and consolidated anti-edema effects) were evaluated compared to those of NF as a whole formulation. A BCCAO model of the renal artery was used to investigate the anti-edema effects of the test samples. The following figures show the results related to a significant increase in vascular permeability (NGL = 1.23 ± 0.03) and, consequently, edema formation after ischemic and RE damage (Figure 5a) compared to basal conditions (before ischemic damage, NGL = 0.11 ± 0.02) (Figure 5b). In the animal-induced edema model used, our results indicate that the i.v. administration of bromelain (1.8 mg/kg b.w.) favored the reduction of extravasation events (NGL = 0.91 ± 0.03, −26.1%, *p* < 0.01 vs. Ctrl), indicating a reduction in capillary permeability and thus an anti-edema effect (Figure 5c,d). Interestingly, i.v. administration of the complete NF (33 mg/kg b.w.) was shown significantly reduce the extravasation phenomenon (NGL = 0.40 ± 0.09, −67.1%, *p* < 0.01 vs. Ctrl), resulting in a valuable reduction in capillary permeability and related edema.

## 3. Discussion

Urolithiasis is a complex pathological syndrome characterized by the presence of calculi at the urinary tract level. The formation of urinary stones is a multifactorial process that can be modulated at each individual step [21]. Clinically, one of the most diffuse pharmaceutical approaches for managing urolithiasis disorders is the MET strategy, which relies on the use of Ca-blockers to induce a urethral smooth muscle relaxation, which in turn could facilitate the passage of ureteral stones. To evaluate the application of the designed NF to support the application of MET, NF was tested on HPASMC cells to investigate the inhibitory activity on muscle contractility, as determined by [Ca^2+^]_i_ levels. Our results showed that treatment with NF resulted in a—49.4% reduction in smooth muscle contractility by (*p* < 0.01 vs. Ctrl), whereas treatment with the monocomponent OrE instead resulted in a less significant reduction in muscle contractility (−15.0%, *p* < 0.05 vs. Ctrl). Origanum vulgare L is an important herbal remedy traditionally employed for its anti-urolithic effects and especially for its peculiar anti-spasmolytic activity [22]. For these reasons, it was used as a reference sample in our experimental protocol. Since in the experimental system used HPASMC contraction was caused by the activation of muscarinic receptors, mainly of the M3 subtype, induced by Ach treatment, the decreased [Ca^2+^]_i_ levels observed after treatment with OrE and NF could be related to a potential inhibitory activity of these samples on muscarinic receptors.

As regards OrE-based treatment, a similar result was also found by other authors who observed that Origanum aqueous extract was able to block smooth muscle contraction via two possible metabolic pathways, such as inhibition of the muscarinic receptor and L-type voltage-dependent calcium channels [22]. Furthermore, based on the greater [Ca^2+^]_i_ reduction obtained after treatment with NF (tested at the same concentration of OrE component), a potential synergistic activity of the NF components could be hypothesized in relation to the beneficial myorelaxant effect observed in vitro. Recently, Esie and colleagues reported that an extract obtained from Ortica leaves attenuated the contractions elicited by electrical field stimulation at all frequencies in a rat’s prostate smooth muscle [23]. Similarly, they also found that the contractions induced by exogenous administration of adenosine triphosphate (ATP) and α,β-methylene-ATP were inhibited by the tested extract, while those induced by stimulation with noradrenaline or Ach were completely unaffected [23,24]. Overall, these observations reasonably suggest the combination of OrE and OE in a unique NF, as they may act synergically through different and complementary mechanisms of action in lowering [Ca^2+^]_i_ levels and, consequently, HPASMC contraction.

According to the MET approach, myorelaxant agents are often combined with anti-edema drugs [25]. Based on this consideration, we aimed to investigate the anti-edema effects of the whole NF compared to only the single bromelain component. Our results showed that treatment with the bromelain-based monocomponent resulted in a significant reduction in vascular permeability and associated edema in a BCCAO model induced in rats. In this regard, the anti-edema effect of bromelain is extensively reported in the scientific literature both in vitro and in vivo [26]. However, the greater significant reduction in vascular permeability and edema formation observed after NF treatment may be due to additional anti-edema effects of other constituents contained in the NF sample. In particular, some authors reported a remarkable anti-edema effect exerted after treatment with an Ortica ethanolic extract in a rats-carrageenan paw edema model [27]. Furthermore, scientific data provide further evidence about the beneficial role ascribed to treatment with the Ortica-based extract in patients with cardiac insufficiency. Specifically, supplementation with this extract resulted in a significant increase in excreted urine volume by 1000 mL, which ameliorated the edema status of these patients [27].

Another therapeutic strategy commonly used in litholysis prophylaxis is related to the use of so-called “stone inhibitors” (SI), which include alkalis (e.g., K and Mg citrate) and the “stone breaker” plant extracts (e.g., extracts of PN and CE). According to our experimental protocol, the ability of the complete NF compared to the SI mix alone (Mg and K citrate, PN, and CE) to inhibit CaOx stone formation was investigated in an in vitro system. The results obtained showed the higher inhibitory effect of NF after both pre-treatment and treatment incubation, resulting in a −52.3% and 70.8% reduction in CaOx crystal formation, respectively, compared to the control. The superiority of NF was also evident when compared to treatment with the single SI mixture (−50% and −15% vs. Ctrl, respectively, for pre-treatment and treatment incubation protocol). Once again, the observed results could be related to the effectiveness of NF in inhibiting the formation and growth CaOx crystals due to the combination of different functional ingredients. Specifically, evidence from the scientific literature reported the ability of an Ortica butanolic extract rich in coumarin glycosides to inhibit the formation of kidney CaOx stones in rats [28]. Moreover, other authors reported that the aqueous extract of the same plant matrix could modulate the morphology and structure of CaOx crystals in an in vitro system [29]. Nevertheless, OrE has also shown a valuable effect in preventing the formation of CaOx stones [22], while bromelain treatment does not seem to have any documented activity in the modulating CaOx crystals.

Hyperuricemia is a pathological condition characterized by high plasma UA concentrations. This condition is closely related to urolithiasis, as high circulating levels of this molecule are the main predisposing cause of UA-based stone formation [30]. Conventional pharmaceutical therapy for the management of hyperuricemia includes the use of allopurinol- and benzbromarone-based treatments, which are associated with severe and well-documented adverse effects [31]. Previous publications have reported that 70% of urate produced in humans is mainly regulated by the renal urate transport system [20], and in particular, the expression levels of cellular transporters OAT1 and URAT1 have recently been defined as potential therapeutic targets for the treatment of hyperuricemia [11]. Considering this evidence, the effects of NF in comparison to OE, a traditional herbal remedy for the treatment of hyperuricemia, on the expression of OAT1 and URAT1 in the HK2 tubule epithelial cell line were evaluated. Noteworthy, the data obtained showed that the NF tested induced both a significant (*p* < 0.01) up-regulation of OAT1 and down-regulation of URAT1 in HK2 cells. Interestingly, the results presented showed prominent effects of NF compared to the OE reference sample, suggesting that the simultaneous presence of different active agents in the NF complex may contribute to the observed effects. In this regard, although no relevant activity on UA clearance has been reported for PN, CE, bromelain, K, and Mg citrate both in vitro and in vivo, oregano extract has shown remarkable inhibition (85.19%) of xanthine oxidase, a key enzyme involved in UA synthesis [19,32]. Based on these observations, it could be assumed that OE and the OrE contained in the NF sample could act in a complementary manner to lower the intracellular concentration of UA.

## 4. Materials and Methods

### 4.1. Reagents

All the extracts used for the NF preparation were supplied and certified by Anvest Health s.r.l. (Milan, Italy). Specifically, all the plant materials used in the NF, registered as Urexina^®^, are aqueous extracts differently characterized. *Phyllanthus niruri* was titrated in 15% of tannins (*w*/*w*)*, Origanum vulgare* L. was titrated in 1% of saponin, and *Urtica diodica* L. was titrated in 0.8% of β-sitosterol. In addition, the K and Mg citrate were supplied pure at 99.99%, and the bromelain was titrated in 2500 gelatin dissolving units (GDU)/g. Specifically, all these components were combined in order to prepare a potential oral nutraceutical formulation containing 200, 100, 30, 10, 20, 5, and 5 mg of K citrate, Mg citrate, PN, CE, bromelain, OrE, and OE, respectively. In all experimental procedures, the activity of NF was compared to a reference sample whose efficacy has already been reported for each target evaluated (i.e., OE for the effect on UA cellular transporters expression, OrE for the miorelaxing effect, SI mix for the inhibition of CaOx stone formation, and bromelain for the anti-edema activity).

### 4.2. Effect of NF on Uric Acid (UA) Cellular Transporters Expression in Human Renal Tubular Epithelial Cells (HK2)

#### 4.2.1. Cell Culture and Toxicity

Human renal tubular epithelial (HK2) cell lines were purchased from Nanjing Cobioer Biotechnology Co., Ltd. (Nanjing, China). All the reagents used for cell culture experiments were purchased from Thermo Scientific (Waltham, MA, USA), otherwise stated. HK2 cells were grown in RPMI 1640 medium composed of 10% fetal bovine serum (FBS), penicillin (110 U/mL), and streptomycin (110 μg/mL) at 37 °C with 5% CO_2_. Stock cultures were grown by replacing the medium every 2 days. The assay was performed when the cells reached approximately 80% of confluence. The toxicity of the NF and OE on HK2 cell lines was assessed using the 3-(4,3-(4,5-dimethylthiazol-2-yl)-2,5 diphenyltetrazolium bromide 5-dimethylthiazol-2-yl)-2, 5-diphenyltetrazolium bromide (MTT) assay. Briefly, HK2 cells were cultured in 96-well plates (0.5 × 10^5^ cells/well) and treated with NF and OE at different concentration levels. Specifically, NF concentrations tested were 37, 74, 370, 555, and 740 μg/mL, while OE was tested at concentrations of 0.5, 1, 5, 7.5, and 10 μg/mL, which correspond exactly to the amount of OE occurring in each NF concentration level tested. Cells were incubated with the NF or OE for 48 h. After this period, the medium was removed and 200 µL of MTT (0.25 mg/mL) was added to each well. After 3 h of incubation, the formazan crystals were dissolved in dimethyl sulfoxide (DMSO) (Merck KGaA, Darmstadt, Germany). The absorbance was measured with a microplate spectrophotometer (Multiskan^TM^ GO microplate spectrophotometer, Thermo Scientific, Waltham, MA, USA) at 570 nm [33].

#### 4.2.2. Cell Treatments and Quantitative Real-Time PCR (RT-qPCR)

In order to evaluate the effects of NF and OE on the expression of urate transporter 1 (URAT1) and organic anion transporter 1 (OAT1), quantitative Real-Time PCR (RT-qPCR) was performed after the treatment experiments. Specifically, HK2 cells (1 × 10^4^ cells/well) were treated with NF (370 μg/mL) or OE (5 μg/mL) for 30 min before being stimulated with UA (Sigma-Aldrich, St. Louis, MO, USA) for 6 h. The control was performed by treatment with an equal volume of the cellular medium. After this incubation period, the total RNA of HK2 cells was extracted using TRI-Reagent (Sigma-Aldrich, Milan, Italy), according to the manufacturer’s instructions, followed by reverse-transcription using iScript^TM^ Reverse Transcription Supermix (Bio-Rad, Milan, Italy). RT-PCR was performed using CFX384 real-time PCR detection system (Bio-Rad, Milan, Italy). Relative gene expression was obtained by normalizing the C^t^ values of each experimental group against β-actin transcript level, using the ^2−^∆Ct formula. mRNA levels were expressed as arbitrary units (A.U.). Primer sequences were designed according to the previously reported protocol [34]:

URAT1: 5′-GCTACCAGAATCGGCACGCT-3, 3′-CACCGGGAAGTCCATCC-5′

OAT1: 5′-ACGGGAAACAAGAAGAGGG-3′, 3′-AAGAGAGGTATGGAGGGGTAG-5′.

### 4.3. Miorelaxing Effects of NF on HPASMC

#### 4.3.1. HPASMC Culture and Toxicity

Human pulmonary artery smooth muscle cells (HPASMC, Thermo Fisher Scientific, Monza, Italy) were used to perform the assay. These are primary cell lines cryopreserved at the end of tertiary culture. Cells were cultured in smooth muscle cell medium (SMCM) (ScienCell, Carlsbad, CA, USA), in 96-well plates pre-stratified with 0.1 mg/mL poly-L-lysine (PLL) (Sigma-Aldrich, Milan, Italy) and placed in an incubator humidified with air at 5% CO_2_, at a temperature of 37 °C. The medium was changed every 2–3 days. To evaluate the cell toxicity of OrE and NF on HPASMC, the MTT assay was performed similarly to what was previously described. Precisely, cells were plated at the concentration of 1 × 10^4^ cells/well into 96-well plates and treated with different concentrations of both NF (37, 74, 370, 555, and 740 μg/mL) and OrE (0.5, 1, 5, 7.5, and 10 μg/mL). After this time, 200 µL of the MTT reagent was added to each well and the multi-well plates were incubated in a humidified atmosphere (i.e., 37 °C, 5% CO_2_) for 48 h. After this time, the formazan crystals were solubilized in DMSO. Absorbance was measured by using a microplate spectrophotometer at 570 nm [35].

#### 4.3.2. Cell Treatment and Determination of [Ca_2_^+^]_i_

In order to evaluate the potential myorelaxant effect, the intracellular Ca concentration [Ca_2_^+^]_i_ was determined by fluorimetric assay. For this purpose, HPASMC were placed on a confocal dish (MatTek, Ashland, MA, USA) and incubated with fluo-3/acetoxymethyl (AM) (5 μM; Thermo Fisher Scientific, Massachusetts, USA) for 30 min at room temperature. After washing with Ca^2+^ containing buffer (160 mM NaCl, 5 mM CaCl_2_, 2.5 mM KCl, 6H_2_O, 1 mM MgCl_2_, 10 mM HEPES, and 8 mM glucose, pH 7.3) [36], the dish was placed on the LSM 510 (ZEISS, Oberkochen, Germany) and buffer flowed continuously during the experiment at a flow rate of 1 mL/min, 23 °C. To determine the Ca influx into the cells, the cell culture was stimulated with acetylcholine (Ach) at the concentration of 10^−5^ mM for 10 s, and then perfused with the Ca^2+^-containing buffer (control). The same experiments were performed by pre-incubating for 30 min the cells with NF (370 μg/mL) or OrE (5 μg/mL) before ACh stimulation. Fluorescence generation was measured using a fluorescent microplate reader (excitation 488 nm and emission 505 nm; GloMax^®^-Multi Detection System, Promega, Milan, Italy).

### 4.4. In Vitro NF Effects on CaOx Crystal Formation and Dimension

#### In Vitro Inhibition of CaOx Stone Formation

A synthetic human urine sample was prepared in the laboratory with the following composition: 96% water, 20% urea, 10% sodium chloride, and 10% nitrogen. The pH was set to 5.7, which was frequently observed in the first-morning urine of patients affected by urolithiasis disorders. Precipitation of calcium oxalate crystals was induced by adding 3 mL of 0.1 M sodium oxalate in 50 mL of urine (equivalent to 0.0402 g), after 60 min of incubation, under gentle agitation at 37 °C (human internal temperature) (control). The prepared urine sample was divided into three aliquots, the first of which served as a control (crystallization without test treatments, induced according to the condition explained above), while in the other aliquots, CaOx crystallization was induced in the presence of the SI mix (340 µg/mL) as reference sample or NF (370 μg/mL urine). All treatments were added to the urine 30 min after the precipitation process. Furthermore, to evaluate the preventive effect of NF and only of SI components, the same experiments were repeated by adding both NF and SI mix to the urine sample 30 min before precipitation. After the incubation period, the samples were centrifuged at 3000 rpm for 10 min and the supernatant was removed. Finally, the height pellet obtained was measured.

### 4.5. Anti-Edema Effects of NF

The anti-edema effect of NF and bromelain (as reference compound) was tested on the transient bilateral common carotid artery occlusion (BCCAO) and reperfusion (RE)-related edema induced in rats. Specifically, 12 male rats (Charles River, Calco, Italy) aged approximately 4 weeks were used to investigate the anti-edema effects of treatment with a single bromelain component compared to treatment with NF. All animals were grown in a controlled environment with constant temperature (24 ± 1 °C) and humidity (60 ± 5%) and were subjected to an artificial circadian cycle of 12 h day/night with free access to food and water. The rats were treated according to the guidelines established by the Guide for the Safety and Use of Laboratory Animals (NIH publication 68–23 revised in 1985) and the Local University Ethics Committee and the Ministry of Health (authorization n. 156/2017-PR). According to our experimental protocol, four rats were subjected to BCCAO and RE damage without being treated with the test samples, four rats were subjected to BCCAO and treated with bromelain, while the last four rats were subjected to BCCAO and NF treatment.

The animals were prepared according to the protocols described previously [37]. Specifically, anesthesia was induced with α-chloralose at the concentration of 50 mg/kg body weight (b.w.), intraperitoneally (i.p.) plus urethane (600 mg/kg b.w., i.p.), and maintained with urethane alone (100 mg/kg b.w., i.p., every hour). Rats were tracheotomized, paralyzed with tubocurarine chloride (1 mg/kg b.w., i.p.), and mechanically ventilated with room air and supplemental oxygen as previously reported) [37]. Briefly, the right and left common carotid arteries were isolated for successive clamping. A catheter was placed in the left femoral artery to measure arterial blood pressure, and in the right femoral vein for injection of fluorescent tracers [fluorescein isothiocyanate bound to dextran, molecular weight 70 KDa (FD 70), 50 mg/100 g b.w., intravenously (i.v.), as 5% *wt/vol* solution]. Blood gas measurements were performed on arterial blood samples (ABL5; Radiometer, Copenhagen, Denmark). Throughout all experiments, mean arterial blood pressure, heart rate, respiratory CO_2_, and blood gas values were recorded and kept stable within physiological ranges. Rectal temperature was monitored and preserved at 37.0 ± 0.5 °C, as previously reported. NF and bromelain solutions were obtained by dissolving 33 mg/kg b.w. NF solution and 1.8 mg/kg b.w. bromelain in a saline solution and infused intravenously 10 min before BCCAO and at the beginning of RE. These amounts were determined considering a daily dose in humans of NF (370 mg/day) and the correspondent bromelain content in NF (20 mg/day), followed by the application of the human–animal dose conversion [38]. The increase in permeability was calculated and reported as normalized gray levels (NGL): NGL = (I − Ir)/Ir, where Ir is the average baseline gray level at the end of the vessel filling with fluorescence (average of five windows located outside the blood vessels, using the same windows throughout the experimental procedure), and I is the same parameter at the end of BCCAO or RE. The gray levels were obtained using the MIP image program by averaging data derived from five windows of 50 × 50 mM (10 × objective) and located outside the venules. A computer-assisted device for XY movement of the microscope table was used to localize the same regions of interest.

### 4.6. Statistical Analysis

For all experiments performed, the mean of at least four replicates was calculated for each condition, with the data expressed as mean ± standard deviation (S.D.), otherwise stated. The results were statistically analyzed using a one-way analysis of variance (ANOVA) followed by Dunnett’s post hoc test or Tukey’s multiple comparison test (PRISM software package, Version 8, GraphPad Software Inc., San Diego, CA, USA).

## 5. Conclusions

The multicomponent NF tested in the present work demonstrated high potential in both the prevention and treatment of urolithiasis. In particular, NF was able to statistically decrease the formation and growth of CaOx crystals and UA uptake in HK2 cells, which in turn may lead to a reduction of UA-based stone formation. In addition, NF has shown remarkable applicability in MET therapy, as it showed relevant anti-edema and myorelaxant effects both in vitro and in an animal model system. Overall, in virtue of the simultaneous presence of different active ingredients, NF could be proposed as an effective, natural, and safe integrative remedy that could provide useful support to the conventional pharmacological therapy for the management of urolithiasis. Undoubtedly, further studies are necessary to deepen the chemical composition of every single extract used in order to identify the main molecular component responsible of the biological effects observed.

## Data Availability

The data used to support the findings of this study are included within the article.
